# Associations of dietary iron intake with cardiovascular disease risk and dyslipidemia among Chinese adults

**DOI:** 10.1186/s12944-024-02058-4

**Published:** 2024-03-02

**Authors:** Min Cui, Hao Wu, Hanmo Zhang, Liping Wei, Xin Qi

**Affiliations:** 1 School of Medicine, Nankai University, Tianjin, China; 2 Department of Cardiology, Tianjin Union Medical Center, Tianjin, China

**Keywords:** Iron intake, Cardiovascular disease risk, Restricted cubic spline, Dyslipidemia

## Abstract

**Background:**

Whether iron intake can affect cardiovascular disease (CVD) and dyslipidemia is controversial. However, few studies have focused on reducing the risk of CVD in people at risk for dyslipidemia. This study explored the linear relationship and possible nonlinear relationship between CVD and dyslipidemia.

**Methods:**

Dietary data were obtained from the China Health and Nutrition Survey between 2004 and 2015. The survey included 8173 participants older than 18 years. CVD risk was estimated by the Framingham risk score (FRS). Logistic regression analysis was used to determine whether iron intake affects CVD incidence and lipid profiles. The nonlinear association was tested with restricted cubic splines (RCSs).

**Results:**

For males, higher total iron intake [the fifth quintile (Q) vs. Q1 odds ratio (OR): 0.335, 95% confidence interval (CI): 0.248–0.453], heme iron intake (OR: 0.679, 95% CI: 0.492–0.937) and non-heme iron intake (OR: 0.362, 95% CI: 0.266–0.492) reduced CVD incidence. Heme iron intake increased high low-density lipoprotein cholesterol (LDL-C) (OR: 1.786, 95% CI: 1.226–2.602), high total cholesterol (TC) (OR: 2.404, 95% CI: 1.575–3.669), high triglyceride (TG) (OR: 1.895, 95% CI: 1.423–2.523), and low apolipoprotein A1/apolipoprotein B (ApoA-1/ApoB) risk (OR: 1.514, 95% CI: 1.178–1.945). Moderate non-heme iron intake reduced high-density lipoprotein cholesterol (HDL-C) incidence (Q5 vs. Q1 OR: 0.704, 95% CI: 0.507–0.979). For females, higher total iron intake (Q5 vs. Q1 OR: 0.362, 95% CI: 0.266–0.492) and non-heme iron intake (OR: 0.347, 95% CI: 0.154–0.781) reduced CVD incidence. Heme iron intake increased high LDL-C (OR: 1.587, 95% CI: 1.160–2.170) and high TC incidence (OR: 1.655, 95% CI: 1.187–2.309).

**Conclusions:**

Men, especially those at risk of developing dyslipidemia, should consume non-heme rather than heme iron to reduce CVD incidence. For women, increased heme iron intake did not reduce CVD incidence. Therefore, women should minimize their heme iron intake to prevent dyslipidemia.

**Supplementary Information:**

The online version contains supplementary material available at 10.1186/s12944-024-02058-4.

## Background

Iron is involved in the formation of hemoglobin and many other functions. However, excessive intake of iron may pose some health risks. Free iron can destroy cellular macromolecules through the Fenton and Haber-Weiss reactions and promote cell death and tissue damage [1]. However, whether iron intake can affect cardiovascular disease (CVD) and dyslipidemia remains inconclusive. A Swedish study showed that consuming more heme iron promoted fatal acute myocardial infarction [2]. Women who consumed more heme iron were eager to develop coronary heart disease [3]. Chen et al. [4] showed that moderate dietary iron intake can prevent nonfatal CVD. However, these studies did not reach a uniform conclusion. In addition, these studies examined only individual cardiovascular diseases and end events and did not cover other related cardiovascular diseases. Therefore, further exploration is warranted.

Dyslipidemia is essential for the development of CVD [5]. However, whether iron intake can influence dyslipidemia is still controversial. A study from Brazil showed that eating more heme iron increased triglyceride levels [6]. A study assessing health and nutritional status showed that iron intake increased triglycerides in women [7]. In addition, few studies have examined whether iron intake influences lipoprotein (a) (Lp(a)) levels and apolipoprotein A1 (ApoA-1)/ apolipoprotein B (ApoB) levels. Therefore, it is necessary to explore whether iron intake influences different lipid markers, including ApoA-1/ApoB and Lp(a), and to explore possible nonlinear relationships.

People in Western countries tend to have a diet centered around meat consumption, while people in China tend to have a predominantly plant diet. Research has indicated that heme iron contributes to 4% of total iron intake in China [8], and 10–15% in Western countries [9]. However, how iron intake affects blood lipids in China remains unknown. In addition, how to take into account the risk of other diseases while reducing the risk of CVD is also a problem that needs to be solved, but few studies have explored this topic. This study is the first to investigate whether iron intake can affect CVD risk and dyslipidemia in China and explore possible nonlinear relationships to prevent the development of CVD and dyslipidemia.

## Methods

### Study population

Data from the China Health and Nutrition Examination Survey (CHNS) were collected. The CHNS is a cohort study in China aimed at investigating nutritional status [10]. The study population came from across China. A multistage random cluster sampling method was used to extract samples.

**Fig. 1:**
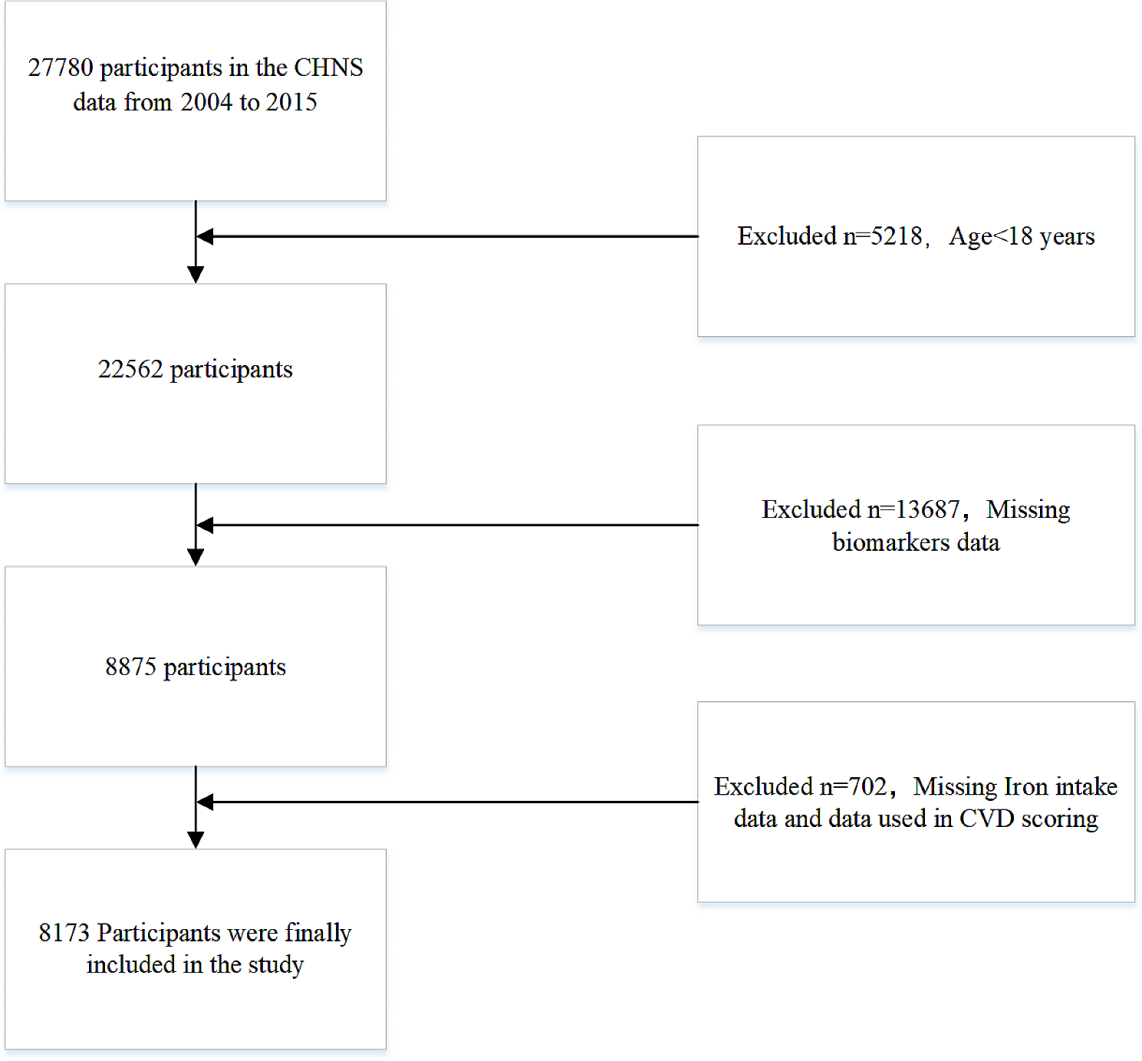
Flowchart of the participant selection process

Due to changes in Chinese food coding before and after 2004, data from only 2004 to 2015 were analyzed in this study. This study involved 27,780 participants. After excluding 5218 participants under 18 years old, 13,687 participants without blood test data, 16 participants without dietary intake data, 649 participants without blood pressure test data, and 38 participants lacking lipid data, a total of 8173 participants (3780 males and 4393 females) were ultimately analyzed. (Fig. 1). A comparative analysis of the general information between the included and excluded adult participants was also conducted to uncover and minimize selection bias (Supplementary Materials, Table S1).

### Laboratory data

Total cholesterol (TC), triglyceride (TG), high-density lipoprotein cholesterol (HDL-C), and low-density lipoprotein cholesterol (LDL-C) were determined via enzymatic methods. ApoB, ApoA-1, and Lp(a) were determined by immunoturbidimetry (Randox, UK). The soluble transferrin receptor (TRFR) and transferrin (TRF) were determined via nephelometry (Siemens, Germany). ferritin (FER) levels were determined by radioimmunology (Bio-Tech, China). The details of the laboratory analysis are reported in CHNS [11].

### Assessment of dietary intake

Diet assessments were conducted using weighed food stocks and three consecutive 24-hour meal recalls. A study assessed the accuracy of 24-hour meal recalls compared to food inventory weights and the difference was 1% (74 kcal/day) [12]. Another study evaluated 24-hour dietary recall and revealed no significant differences with weighed dietary records, indicating good consistency [13]. Heme iron accounts for approximately 40% of the iron in meat [14], including fish, poultry and livestock. After logarithmic conversion, they were adjusted for energy by residual method [15].

### Assessment of CVD risk

The Framingham risk score (FRS) was used to estimate the risk of developing CVD [16]. The FRS was calculated based on sex, age, smoking status, TC, HDL-C, systolic blood pressure (hypertension treatment and systolic blood pressure values), and diabetes status, and ≥ 20% was considered high risk [17, 18].

### Definitions of dyslipidemia

High TC levels were defined as TC ≥ 6.2 mmol/L (240 mg/dL). High TG levels were defined as TG ≥ 2.3 mmol/mol (200 mg/dL). Low HDL-C levels were defined as HDL-C < 1.0 mmol/L (40 mg/dL); high LDL-C levels were defined as LDL-C ≥ 4.1 mmol/L (160 mg/dL). Low ApoA-1/ApoB levels were defined as ApoA-1/ApoB < 1, and high Lp (a) levels were defined as Lp (a) > 300 mg/L. The presence of any abnormality in lipid biomarkers is considered to indicate dyslipidemia [19].

### Assessment of covariates

General and lifestyle information, including age, sex, residential area, educational level, smoking status, alcohol consumption, and occupation (farmer, worker, other), was collected using a structured questionnaire. Hypertension and diabetes were self-reported diagnoses. Blood pressure was averaged over three measurements. Body mass index (BMI) was calculated as weight (kg) divided by the square of height (m).

### Statistical analyses

The differences between groups were compared by ANOVA, the Mann‒Whitney U test, or the chi-square test. A logistic regression model was used to calculate odds ratios (ORs) and 95% confidence intervals (CIs). Model 1 was unadjusted. Model 2 was adjusted for BMI, alcohol status, energy intake, urban residence, and education level. Model 3 was further adjusted for serum ferritin, transferrin, and transferrin receptor levels. For the dyslipidemia risk model, Model 1 was unadjusted, and the adjusted model accounted for age, BMI, alcohol status, smoking status, energy intake, urban residence, education level, and serum ferritin, transferrin, and transferrin receptor levels. All covariates were known potential risk factors for CVD incidence, dyslipidemia or diet, and biochemical confounders. There is no apparent multicollinearity. The rms packet was used to fit the restricted cubic spline (RCS). Harrell suggested that the model fit well with four knots, striking a balance between the smoothness of the curve and avoiding overfitting that may result in reduced accuracy [20]. The 5th, 35th, 65th, and 95th percentiles were used as knots. Sensitivity analysis was performed by excluding patients with hypertension and diabetes, patients diagnosed with myocardial infarction, or patients with a BMI < 18 kg/m^2^ at baseline. The information on missing values is presented in the Supplementary Materials, Table S2. Missing values were imputed using the median. R software (version 4.1.0) was used for statistical analysis. *P* < 0.05 was considered significant.

## Results

### Baseline characteristics

This study included 8173 participants (Table 1, Supplementary Materials, Table S3 and Table S4). The mean age was 47.3 years at baseline. A total of 46.2% were males. The average BMI was 22.1 kg/m^2^. People who consumed more heme iron were mostly men, were younger, had lower blood pressure, had higher education levels, were more urban residents, had a greater history of alcohol consumption, and were fewer farmers and more workers at baseline. People who consumed more non-heme iron were younger, had lower blood pressure, had a greater history of alcohol consumption; were fewer urban residents, had lower education levels, and had a greater proportion of farmers. As heme iron intake increased, fat intake increased and carbohydrate intake decreased while serum ferritin increased, transferrin decreased, and TC and TG levels increased. As non-heme iron intake increased, energy, carbohydrate, and protein intake increased, while serum ferritin increased, transferrin decreased and LDL-C decreased.

**Table 1:** Population characteristics by quintiles of dietary heme and non-heme iron intake

Variables*	Quintiles of heme iron intake,mg/day				Quintiles of non-heme iron intake,mg/day			
	Q1	Q3	Q5	P	Q1	Q3	Q5	P
Male, n(%)	658(40.2)	709(43.4)	904(55.3)	< 0.001	469(28.7)	784(48.0)	1003(61.3)	< 0.001
Age, years	49.0(38.0,60.0)	47(37.0,58.0)	46(35.5,54.0)	< 0.001	52.0(39.0,65.0)	47.0(37.0,56.0)	45.0(37.0,54.0)	< 0.001
Body mass index, kg/m^2^	21.8(20.1,24.2)	21.7(20.0,24.0)	21.5(19.8,24.0)	< 0.001	21.9(20.0,24.4)	21.8(20.0,23.9)	21.6(19.9,23.7)	0.103
Systolic BP, mmHg	116.7(105.0,124,7)	112.0(104.7,122.0)	110.7(104.0,120.7)	< 0.001	118.0(106.7,129.3)	112.0(104.5,121.7)	111.3(104.7,120.7)	< 0.001
Diastolic BP, mmHg	76.0(70.0,80.5)	75.0(68.7,80.0)	74.0(69.3,80.0)	< 0.001	76.7(70.0,82.0)	75.0(69.3,80.0)	75.0(70.0,80.0)	< 0.001
Smoke, n(%)	355(21.7)	320(19.6)	356(21.8)	0.440	298(18.2)	381(23.3)	361(22.1)	0.003
Drinking Alcohol, n(%)	191(11.7)	256(15.7)	301(18.4)	< 0.001	192(11.7)	271(16.6)	280(17.1)	< 0.001
Diabetes, n(%)	10(0.6)	18(1.1)	16(1.0)	0.244	20(1.2)	16(1.0)	12(0.7)	0.143
Hypertension, n(%)	73(4.5)	72(4.4)	64(3.9)	0.690	95(5.8)	46(2.8)	52(3.2)	< 0.001
Urban Residence, n(%)	260(15.9)	551(33.7)	775(47.4)	< 0.001	577(35.3)	547(33.5)	482(29.5)	0.005
Education level, n(%)				< 0.001				< 0.001
Primary school or lower	1087(66.5)	829(50.7)	635(38.8)		889(54.4)	828(50.6)	763(46.7)	
Middle school	511(31.3)	719(44.0)	904(55.3)		661(40.4)	721(44.1)	802(49.1)	
college or above	9(0.6)	55(3.4)	77(4.7)		55(3.4)	61(3.7)	47(2.9)	
Occupation, n(%)				< 0.001				< 0.001
Farmer	982(60.1)	704(43.1)	463(28.3)		561(34.3)	718(43.9)	791(48.4)	
Worker	181(11.1)	245(15.0)	380(23.2)		292(17.9)	264(16.1)	275(16.8)	
Other	472(28.9)	686(42.0)	792(48.4)		782(47.8)	653(39.9)	569(34.8)	
Dietary intake								
Heme iron, mg/day	0.1(0.0,0.2)	0.8(0.7,0.9)	2.0(1.7,2.6)	< 0.001	0.6(0.3,0.9)	0.8(0.4,1.4)	1.0(0.4,1.6)	< 0.001
Nonheme iron, mg/day	17.3(14.3,21.5)	17.9(14.9,21.3)	20.2(17.4,24.1)	< 0.001	12.8(11.5,13.7)	18.2(17.6,18.9)	26.8(24.7,30.7)	< 0.001
Iron, mg/day	17.5(14.4,21.6)	18.7(15.7,22.1)	22.5(19.5,26.6)	< 0.001	13.5(12.0,14.4)	19.2(18.4,19.9)	28.2(25.8,32.2)	< 0.001
Energy, Kcal/day	2473.2(1925.1,3019.0)	2347.2(1918.7,2810.2)	2387.3(2012.0,2864.4)	< 0.001	2097.1(1648.5,2544.9)	2406.3(2019.2,2866.7)	2677.3(2199.9,3184.6)	< 0.001
Fat, g/day	41.5(28.1,60.7)	61.8(42.7,88.5)	74.9(51.4,100.6)	< 0.001	56.9(38.1,81.6)	60.1(39.4,88.6)	59.7(39.4,90.0)	0.001
Carbohydrate, g/day	427.9(324.1,558.1)	361.0(273.9,446.1)	337.2(269.7,421.1)	< 0.001	316.5(232.9,404.6)	370.4(294.4,465.3)	420.8(327.5,535.3)	< 0.001
Protein, g/day	69.2(53.0,88.1)	67.4(54.3,83.3)	74.7(62.2,91.1)	< 0.001	60.2(47.4,75.3)	71.4(57.7,86.1)	77.9(64.1,96.4)	< 0.001
Biomarkers								
Ferritin, ng/ml	74.0(38.0,133.6)	75.1(37.7,143.0)	94.9(46.4,171.1)	< 0.001	69.3(32.5,128.0)	81.0(43.3,146.9)	89.1(45.9,155.8)	< 0.001
Transferrin, mg/dl	287.5(257.0,324.0)	282.0(248.0,318.0)	280.0(250.8,314.0)	< 0.001	279.0(250.0,316.0)	282.0(252.0,316.0)	283.0(252.0,320.0)	0.152
Transferrin receptor, mg/L	1.3(1.1,1.6)	1.4(1.1,1.7)	1.3(1.1,1.6)	0.082	1.4(1.1,1.7)	1.4(1.1,1.6)	1.3(1.1,1.6)	< 0.001
LDL-C, mmol/L	2.9(2.3,3.5)	2.9(2.3,3.5)	3.0(2.4,3.6)	0.059	3.0(2.3,3.7)	2.9(2.3,3.5)	2.9(2.4,3.5)	< 0.001
HDL-C, mmol/L	1.4(1.2,1.6)	1.4(1.2,1.6)	1.4(1.1,1.6)	< 0.001	1.4(1.2,1.7)	1.4(1.1,1.6)	1.4(1.2,1.7)	0.006
Total cholesterol, mmol/L	4.7(4.1,5.4)	4.8(4.2,5.4)	4.9(4.3,5.6)	< 0.001	4.8(4.2,5.5)	4.8(4.2,5.5)	4.8(4.2,5.4)	0.025
Triglycerides, mmol/L	1.2(0.8,1.9)	1.2(0.8,2.0)	1.3(0.9,2.1)	< 0.001	1.3(0.9,1.9)	1.3(0.9,2.0)	1.2(0.8,1.9)	0.109
Apolipoprotein A1, g/L	1.07(0.93,1.24)	1.09(0.94,1.28)	1.12(0.96,1.35)	< 0.001	1.10(0.95,1.31)	1.10(0.95,1.29)	1.09(0.93,1.28)	0.009
Apolipoprotein B, g/L	0.85(0.71,1.03)	0.88(0.73,1.06)	0.92(0.76,1.12)	< 0.001	0.90(0.74,1.09)	0.88(0.72,1.07)	0.88(0.73,1.07)	0.021
Lipoprotein (a), mg/L	92.0(47.0,190.0)	76.0(41.0,170.0)	68.0(33.0,147.0)	< 0.001	85.0(45.0,180.5)	83.0(41.0,174.5)	72.0(38.0,151.0)	< 0.001

*Variables are presented as the mean (SD), median (IQR) or n (%)

### Associations between iron intake and high CVD risk

**Fig. 2:**
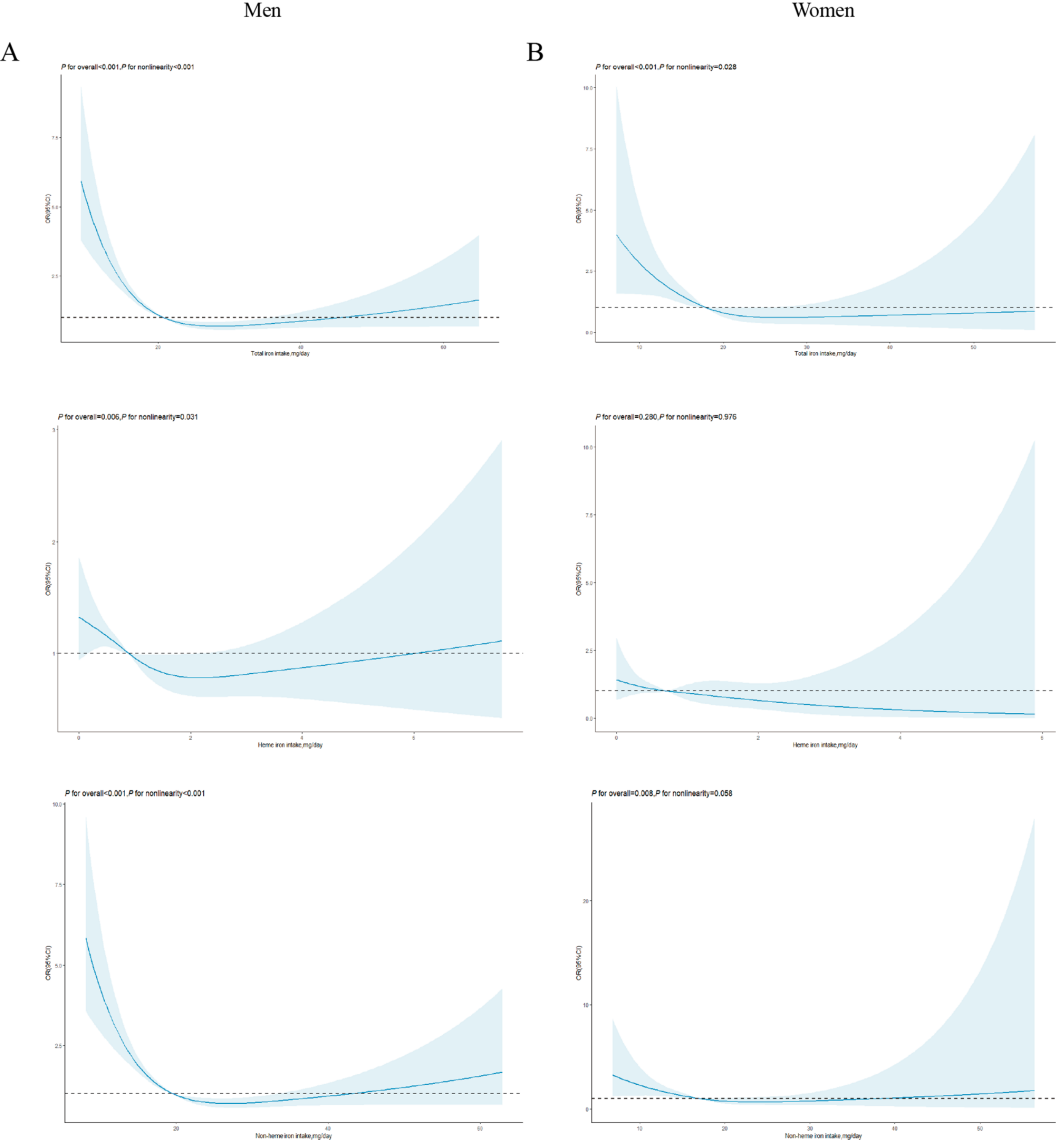
High CVD risk according to iron intake in men (**A**) and women (**B**) in Model 3

For males, higher total iron intake (the fifth quintile(Q) vs. Q1 OR: 0.335, 95% CI: 0.248–0.453, Table 2), heme iron intake (OR: 0.679, 95% CI: 0.492–0.937) and non-heme iron intake (OR: 0.362, 95% CI: 0.266–0.492) reduced the risk of CVD. The RCS analysis revealed an approximately inverse J-shaped relationship between total iron intake (*P* for nonlinearity < 0.001, Fig. 2A) and non-heme iron intake (*P* for nonlinearity < 0.001) and CVD risk, while a U-shaped relationship was shown for heme iron intake (*P* for nonlinearity = 0.031).

**Table 2:** Association between iron intake and high cardiovascular disease risk in men

	Model 1			Model 2			Model 3		
Quintiles of total iron intake	OR	95%CI	*P*	OR	95%CI	*P*	OR	95%CI	*P*
Q1	Ref			Ref			Ref		
Q2	0.541	0.426–0.688	< 0.001	0.564	0.438–0.728	< 0.001	0.569	0.441–0.736	< 0.001
Q3	0.347	0.367–0.451	< 0.001	0.381	0.289–0.503	< 0.001	0.382	0.288–0.505	< 0.001
Q4	0.250	0.188–0.332	< 0.001	0.251	0.185–0.340	< 0.001	0.259	0.190–0.352	< 0.001
Q5	0.284	0.215–0.374	< 0.001	0.324	0.241–0.436	< 0.001	0.335	0.248–0.453	< 0.001
Quintiles of heme iron intake									
Q1	Ref			Ref			Ref		
Q2	1.139	0.880–1.474	0.324	1.057	0.800-1.395	0.698	1.001	0.756–1.327	0.993
Q3	0.955	0.733–1.245	0.735	0.918	0.690–1.221	0.558	0.854	0.640–1.141	0.286
Q4	0.877	0.670–1.147	0.337	0.921	0.687–1.234	0.580	0.846	0.628–1.139	0.270
Q5	0.645	0.485–0.859	0.003	0.732	0.534–1.004	0.053	0.679	0.492–0.937	0.018
Quintiles of non-heme iron intake									
Q1	Ref			Ref			Ref		
Q2	0.541	0.425–0.688	< 0.001	0.549	0.460–0.768	< 0.001	0.612	0.472–0.793	< 0.001
Q3	0.393	0.304–0.508	< 0.001	0.420	0.319–0.552	< 0.001	0.445	0.337–0.588	< 0.001
Q4	0.260	0.195–0.346	< 0.001	0.275	0.203–0.374	< 0.001	0.297	0.218–0.404	< 0.001
Q5	0.279	0.211–0.369	< 0.001	0.334	0.246–0.453	< 0.001	0.362	0.266–0.492	< 0.001

Model 1: Unadjusted. Model 2: Adjusted for BMI, alcohol status, energy intake, urban residence, and education level. Model 3: Adjusted for Model 2 + serum ferritin, transferrin, and transferrin receptor levels. Heme and non-heme iron intakes were adjusted mutually

For females, higher total iron intake (Q5 vs. Q1 OR: 0.362, 95% CI: 0.266–0.492, Table 3) and non-heme iron intake (OR: 0.347, 95% CI: 0.154–0.781) reduced the risk of CVD. The RCS curves demonstrated an approximate L-shaped relationship (*P* for nonlinearity = 0.028, Fig. 2B).

**Table 3:** Association between iron intake and high CVD risk in women

	Model 1			Model 2			Model 3		
Quintiles of total iron intake									
Q1	Ref			Ref			Ref		
Q2	0.574	0.344–0.959	0.034	0.514	0.294–0.899	0.020	0.541	0.308–0.950	0.032
Q3	0.235	0.117–0.473	< 0.001	0.244	0.115–0.518	< 0.001	0.244	0.115–0.520	< 0.001
Q4	0.283	0.148–0.543	< 0.001	0.329	0.164–0.663	0.002	0.355	0.176–0.715	0.004
Q5	0.211	0.102–0.438	< 0.001	0.274	0.124–0.609	0.001	0.282	0.126–0.629	0.002
Quintiles of heme iron intake									
Q1	Ref			Ref			Ref		
Q2	0.912	0.501-1,660	0.763	0.787	0.410–1.513	0.473	0.777	0.403–1.499	0.452
Q3	1.090	0.614–1.935	0.770	1.213	0.652–2.257	0.542	1.117	0.596–2.092	0.730
Q4	0.778	0.417–1.454	0.433	0.870	0.439–1.726	0.690	0.823	0.413–1.642	0.581
Q5	0.385	0.177–0.837	0.016	0.537	0.232–1.244	0.147	0.498	0.214–1.159	0.106
Quintiles of non-heme iron intake									
Q1	Ref			Ref			Ref		
Q2	0.702	0.425–1.161	0.168	0.687	0.396–1.191	0.181	0.716	0.411–1.247	0.237
Q3	0.127	0.050–0.323	< 0.001	0.130	0.045–0.374	< 0.001	0.134	0.047–0.388	< 0.001
Q4	0.410	0.227–0.742	0.003	0.578	0.305–1.093	0.092	0.641	0.337–1.219	0.175
Q5	0.255	0.126–0.514	< 0.001	0.333	0.149–0.746	0.008	0.347	0.154–0.781	0.011

Model 1: Unadjusted. Model 2: Adjusted for BMI, alcohol status, energy intake, urban residence, and education level. Model 3: Adjusted for Model 2 + serum ferritin, transferrin, and transferrin receptor levels. Heme and non-heme iron intakes were adjusted mutually

### Association between iron intake and risk of developing dyslipidemia in men

**Fig. 3:**
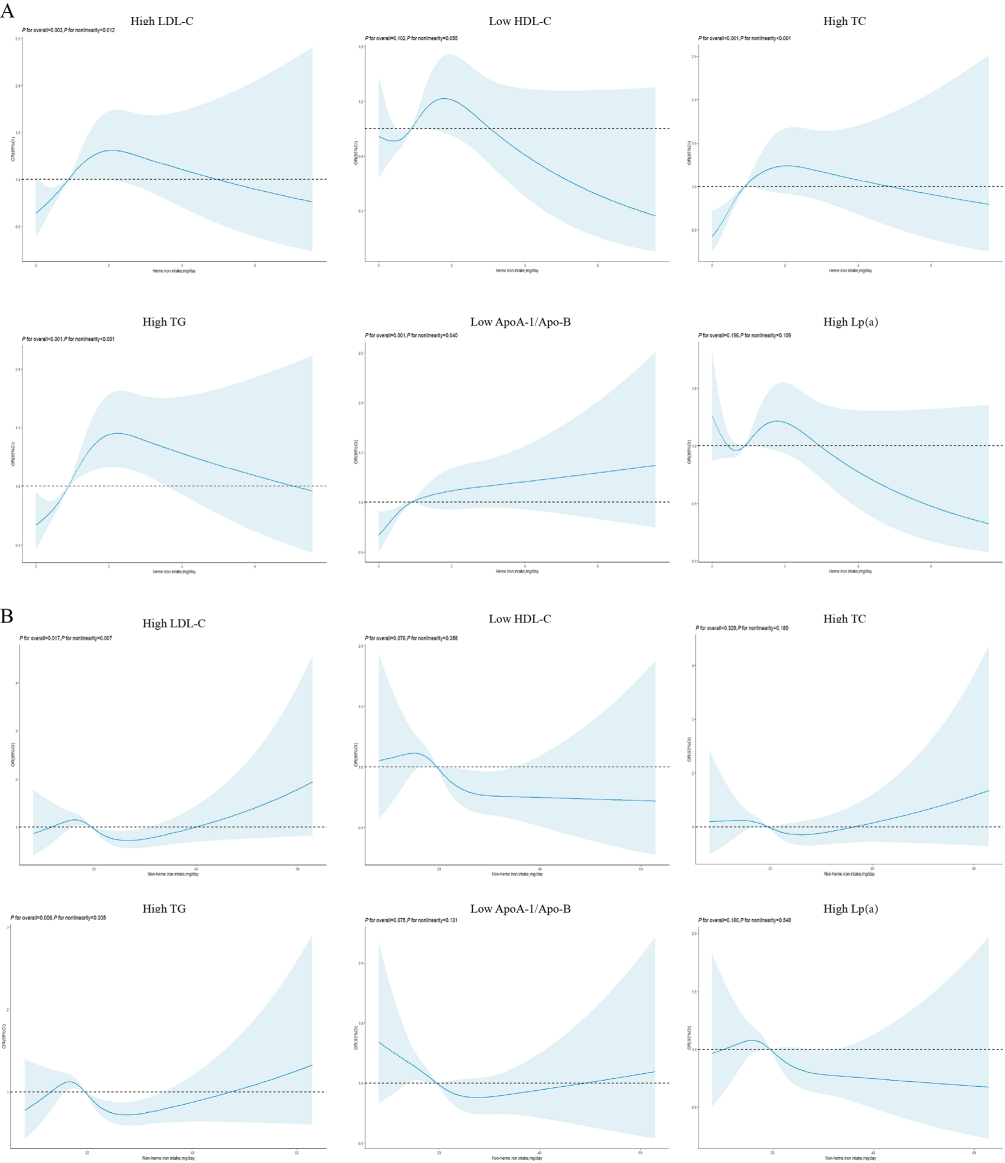
The risk of developing dyslipidemia according to heme (**A**) and non-heme iron intake (**B**) in men in the adjusted model

More heme iron intake increased the high LDL-C levels (Q5 vs. Q1 OR: 1.786, 95% CI: 1.226–2.602, Table 4), high TC levels (OR: 2.404, 95% CI: 1.575–3.669), high TG levels (OR: 1.895, 95% CI: 1.423–2.523), and low ApoA-1/ApoB levels risk (OR: 1.514, 95% CI: 1.178–1.945). Moderate non-heme iron intake reduced low HDL-C levels (Q5 vs. Q1 OR: 0.704, 95% CI: 0.507–0.979), low ApoA-1/ApoB levels (Q4 vs. Q1 OR: 0.742, 95% CI: 0.742) and high Lp (a) levels (Q3 vs. Q1 OR: 0.657, 95% CI: 0.470–0.917). Heme iron intake nonlinearly influenced high LDL-C levels (*P* for nonlinearity = 0.012, Fig. 3A), high TC levels (*P* for nonlinearity < 0.001), high TG levels (*P* for nonlinearity < 0.001), and low apoA-1/apoB levels (*P* for nonlinearity = 0.040), resembling an inverted L-shape. Non-heme iron intake nonlinearly influenced high LDL-C levels (*P* for nonlinearity = 0.007, Fig. 3B) and high TG levels (*P* for nonlinearity = 0.005).

**Table 4:** Association between iron intake and risk of developing dyslipidemia in men

Dyslipidemia Type	heme iron intake						non-heme iron intake					
	Model 1			Adjusted model			Model 1			Adjusted model		
High LDL-C	OR	95%CI	*P*	OR	95%CI	*P*	OR	95%CI	*P*	OR	95%CI	*P*
Q1	Ref			Ref			Ref			Ref		
Q2	1.223	0.842–1.775	0.290	1.194	0.815–1.750	0.362	1.028	0.743–1.421	0.869	1.115	0.797–1.560	0.526
Q3	1.280	0.885–1.952	0.190	1.234	0.841–1.810	0.283	0.946	0.681–1.316	0.743	1.020	0.721–1.443	0.909
Q4	1.593	1.116–2.275	0.010	1.663	1.145–2.416	0.008	0.680	0.476–0.969	0.033	0.702	0.482–1.024	0.067
Q5	1.722	1.212–2.449	0.002	1.786	1.226–2.602	0.003	0.798	0.567–1.124	0.196	0.815	0.559–1.189	0.288
Low HDL-C												
Q1	Ref			Ref			Ref			Ref		
Q2	1.114	0.822–1.509	0.486	1.030	0.749–1.416	0.855	1.000	0.754–1.325	1.000	0.977	0.728–1.312	0.877
Q3	1.140	0.842–1.542	0.397	1.078	0.785–1.481	0.641	0.990	0.746–1.312	0.943	0.914	0.676–1.234	0.556
Q4	1.284	0.955–1.728	0.098	1.183	0.862–1.623	0.298	0.878	0.658–1.172	0.378	0.795	0.582–1.085	0.148
Q5	1.392	1.039–1.866	0.027	1.295	0.942–1.779	0.112	0.704	0.521–0.952	0.023	0.704	0.507–0.979	0.037
High TC												
Q1	Ref			Ref			Ref			Ref		
Q2	1.395	0.920–2.115	0.117	1.538	0.999–2.366	0.050	0.966	0.671–1.391	0.852	0.966	0.659–1.416	0.860
Q3	1.696	1.134–2.536	0.010	1.904	1.248–2.905	0.003	0.983	0.683–1.414	0.926	0.968	0.659–1.422	0.869
Q4	1.696	1.134–2.536	0.010	2.106	1.375–3.226	< 0.001	0.915	0.633–1.324	0.638	0.806	0.541–1.201	0.290
Q5	2.035	1.376–3.011	< 0.001	2.404	1.575–3.669	< 0.001	0.966	0.671–1.391	0.852	0.869	0.579–1.304	0.497
High TG												
Q1	Ref			Ref			Ref			Ref		
Q2	1.047	0.803–1.365	0.735	1.072	0.800−1.435	0.642	1.256	0.982–1.606	0.070	1.142	0.871–1.498	0.338
Q3	1.365	1.058–1.763	0.017	1.570	1.182–2.087	0.002	1.312	1.027–1.676	0.030	1.101	0.837–1.448	0.490
Q4	1.472	1.143–1.896	0.003	1.746	1.314–2.322	< 0.001	1.068	0.831–1.374	0.608	0.794	0.597–1.056	0.113
Q5	1.780	1.389–2.280	< 0.001	1.895	1.423–2.523	< 0.001	1.008	0.782−1.300	0.948	0.787	0.587–1.055	0.109
Low ApoA-1/ApoB												
Q1	Ref			Ref			Ref			Ref		
Q2	1.200	0.951–1.514	0.124	1.219	0.954–1.557	0.113	1.006	0.806–1.256	0.955	0.993	0.786–1.254	0.952
Q3	1.257	0.998–1.584	0.052	1.337	1.046–1.709	0.020	0.962	0.770–1.202	0.733	0.915	0.721–1.160	0.462
Q4	1.429	1.137–1.795	0.002	1.563	1.222–1.999	< 0.001	0.807	0.643–1.013	0.064	0.742	0.580–0.948	0.017
Q5	1.411	1.123–1.774	0.003	1.514	1.178–1.945	0.001	0.907	0.725–1.134	0.392	0.847	0.660–1.085	0.189
High L(a)												
Q1	Ref			Ref			Ref			Ref		
Q2	0.886	0.652–1.202	0.436	0.908	0.664–1.240	0.542	1.134	0.854–1.507	0.385	1.204	0.899–1.611	0.213
Q3	0.874	0.643–1.188	0.390	0.869	0.633–1.192	0.382	0.616	0.447–0.849	0.003	0.657	0.470–0.917	0.013
Q4	1.094	0.815–1.468	0.549	1.105	0.811–1.505	0.527	0.818	0.605–1.106	0.193	0.900	0.656–1.236	0.516
Q5	0.818	0.600−1.117	0.207	0.900	0.647–1.251	0.529	0.767	0.564–1.041	0.089	0.810	0.584–1.122	0.205

Model 1: Unadjusted. Adjusted model: Adjusted for age, BMI, alcohol status, smoking status, energy intake, urban residence, education level, serum ferritin, transferrin, and transferrin receptor level. Heme and non-heme iron intakes were adjusted mutually

### Association between iron intake and the risk of developing dyslipidemia in women

**Fig. 4:**
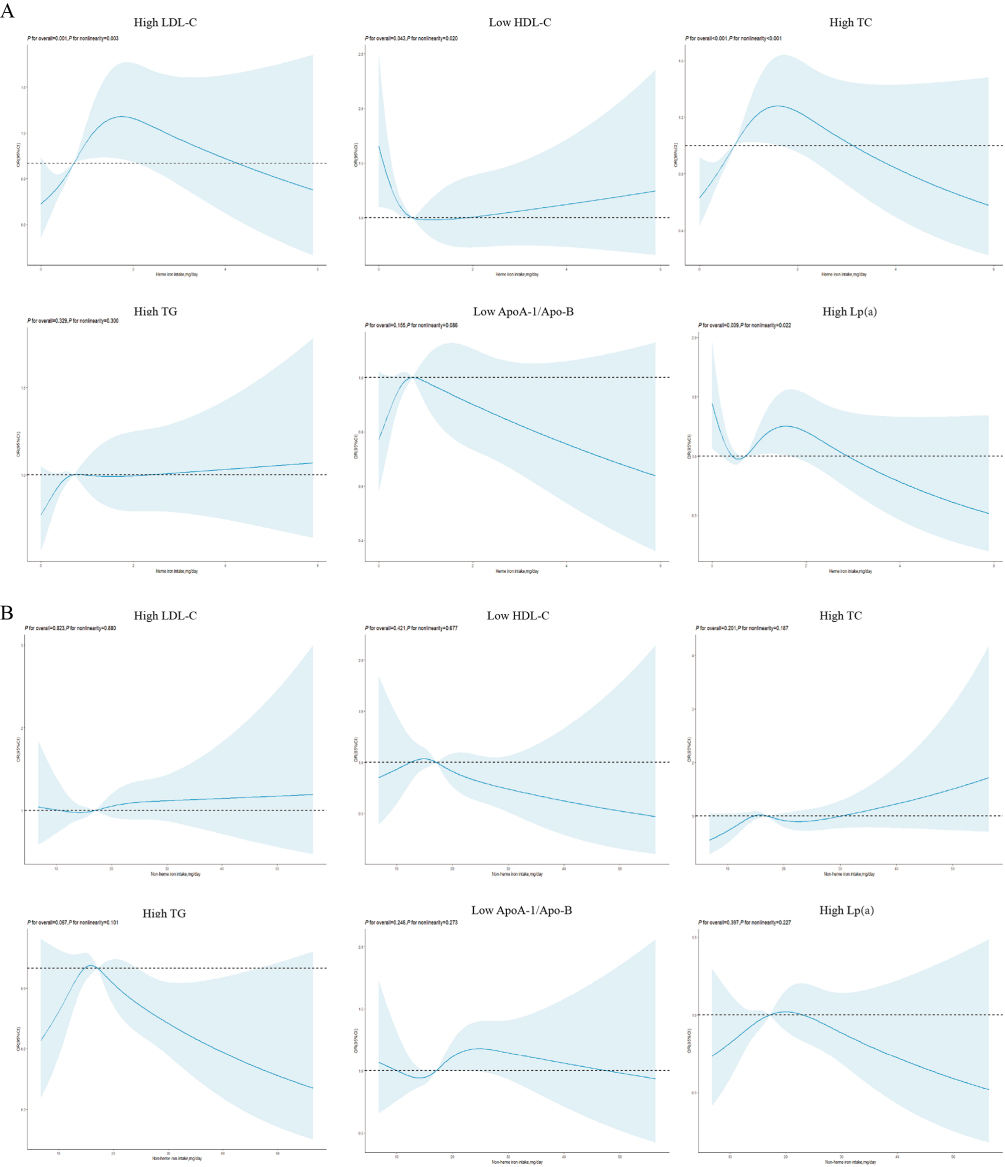
The risk of developing dyslipidemia according to heme (**A**) and non-heme iron intake (**B**) in women in the adjusted model

A higher heme iron intake increased both high LDL-C levels (Q5 vs. Q1 OR: 1.587, 95% CI: 1.160–2.170, Table 5) and high TC level risk (OR: 1.655, 95% CI: 1.187–2.309) after adjustment. Heme iron intake nonlinearly influenced high LDL-C levels (*P* for nonlinearity = 0.003, Fig. 4A), low HDL-C levels (*P* for nonlinearity = 0.020), high TC levels(*P* for nonlinearity < 0.001) and high Lp(a) levels (*P* for nonlinearity < 0.022), and the relationships were similar to an inverted L-shaped curve. Non-heme iron intake was not effective (Fig. 4B).

**Table 5:** Association between iron intake and the risk of developing dyslipidemia in women

Dyslipidemia Type	heme iron intake						non-heme iron intake					
	Model 1			Adjusted model			Model 1			Adjusted model		
High LDL-C	OR	95%CI	*P*	OR	95%CI	*P*	OR	95%CI	*P*	OR	95%CI	*P*
Q1	Ref			Ref			Ref			Ref		
Q2	1.149	0.864–1.530	0.340	1.240	0.918–1.675	0.160	0.901	0.686–1.182	0.451	1.121	0.839–1.497	0.440
Q3	0.978	0.728–1.312	0.881	1.120	0.821–1.529	0.474	0.785	0.593–1.038	0.089	0.970	0.719–1.308	0.840
Q4	1.327	1.004–1.754	0.047	1.664	1.231–2.250	< 0.001	0.803	0.608–1.061	0.122	1.113	0.825–1.503	0.483
Q5	1.206	0.908–1.601	0.195	1.587	1.160–2.170	0.004	0.900	0.685–1.181	0.446	1.228	0.910–1.658	0.179
Low HDL-C												
Q1	Ref			Ref			Ref			Ref		
Q2	0.942	0.670–1.325	0.733	0.846	0.594–1.205	0.354	1.067	0.752–1.515	0.716	1.030	0.717–1.481	0.871
Q3	0.769	0.538–1.098	0.149	0.713	0.492–1.034	0.075	0.935	0.652–1.340	0.714	0.946	0.648–1.381	0.774
Q4	0.812	0.571–1.156	0.248	0.747	0.515–1.085	0.126	0.969	0.678–1.384	0.861	0.998	0.684–1.456	0.993
Q5	0.670	0.463–0.970	0.034	0.674	0.454–1.001	0.051	0.775	0.532–1.128	0.183	0.782	0.522–1.170	0.232
High TC												
Q1	Ref			Ref			Ref			Ref		
Q2	1.090	0.803–1.478	0.581	1.235	0.896–1.702	0.198	0.940	0.707–1.250	0.669	1.156	0.854–1.566	0.347
Q3	1.050	0.773–1.428	0.754	1.264	0.911–1.752	0.160	0.770	0.572–1.036	0.084	0.946	0.690–1.299	0.733
Q4	1.365	1.018–1.829	0.038	1.880	1.369–2.582	< 0.001	0.839	0.627–1.124	0.239	1.148	0.838–1.573	0.390
Q5	1.152	0.852–1.558	0.357	1.655	1.187–2.309	0.003	0.858	0.642–1.147	0.302	1.097	0.797–1.512	0.570
High TG												
Q1	Ref			Ref			Ref			Ref		
Q2	1.049	0.822–1.340	0.700	1.178	0.904–1.534	0.225	0.903	0.705–1.156	0.418	0.876	0.670–1.145	0.332
Q3	0.992	0.775–1.270	0.950	1.203	0.919–1.575	0.178	1.023	0.803–1.304	0.853	1.029	0.790–1.340	0.832
Q4	0.954	0.744–1.223	0.712	1.233	0.938–1.620	0.134	0.895	0.699–1.147	0.382	0.939	0.716–1.231	0.648
Q5	0.846	0.656–1.090	0.197	1.223	0.919–1.626	0.167	0.835	0.650–1.074	0.160	0.811	0.612–1.074	0.143
Low ApoA-1/ApoB												
Q1	Ref			Ref			Ref			Ref		
Q2	1.212	0.968–1.519	0.094	1.287	1.015–1.632	0.037	0.894	0.712–1.121	0.331	0.987	0.776–1.256	0.916
Q3	1.063	0.846–1.337	0.599	1.181	0.925–1.506	0.181	0.855	0.681–1.075	0.181	0.998	0.782–1.274	0.990
Q4	0.981	0.778–1.237	0.869	1.132	0.882–1.453	0.320	0.887	0.707–1.114	0.303	1.117	0.875–1.426	0.376
Q5	0.952	0.754–1.201	0.678	1.153	0.892–1.490	0.276	0.974	0.779–1.219	0.819	1.225	0.957–1.568	0.107
High Lp(a)												
Q1	Ref			Ref			Ref			Ref		
Q2	0.751	0.576–0.978	0.033	0.760	0.580–0.997	0.047	1.321	1.011–1.727	0.041	1.405	1.068–1.850	0.015
Q3	0.772	0.594–1.004	0.054	0.792	0.604–1.039	0.092	1.178	0.897–1.547	0.239	1.288	0.970–1.709	0.080
Q4	0.850	0.657−1.100	0.216	0.905	0.691–1.187	0.472	1.158	0.881–1.522	0.292	1.316	0.989–1.752	0.060
Q5	0.787	0.606–1.023	0.073	0.866	0.654–1.148	0.317	1.062	0.805–1.401	0.671	1.234	0.920–1.657	0.160

Model 1: Unadjusted. Adjusted model: Adjusted for age, BMI, alcohol status, smoking status, energy intake, urban residence, education level, serum ferritin, transferrin, and transferrin receptor level. Heme and non-heme iron intakes were adjusted mutually

### Subgroup and sensitivity analyses

**Fig. 5:**
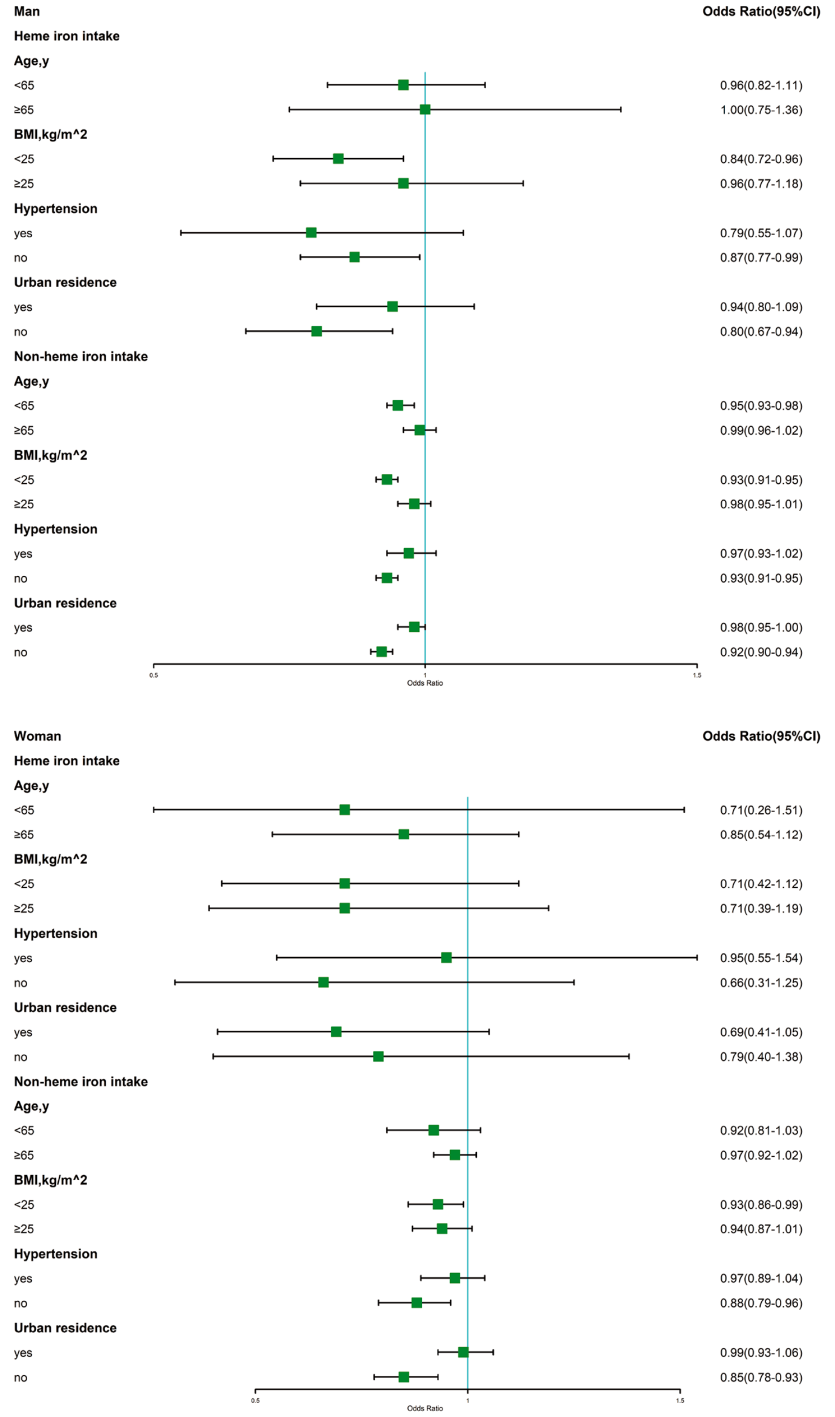
Subgroup analysis, in Model 3 excluding the subgroup variable itself. The OR was calculated per 1-unit increase in heme or non-heme iron intake

Participants were stratified by age (< 65 or ≥ 65 years), BMI (< 25 or ≥ 25 kg/m^2^), hypertension status (yes or no), and urban residence status (yes or no) (Fig. 5). In men, CVD risk decreased in subgroups with a BMI < 25 kg/m^2^, individuals without hypertension, and individuals in the rural resident subgroup. In women, non-heme iron intake reduced CVD risk in subgroups with a BMI < 25 kg/m^2^, no hypertension, and no urban residence, while heme iron intake did not.

Sensitivity analysis was performed by excluding candidates with hypertension and diabetes (Supplementary Materials, Table S5), patients diagnosed with myocardial infarction (Supplementary Materials, Table S6), or candidates with a BMI < 18 kg/m^2^ at baseline (possible digestive malabsorption affecting iron digestion and absorption) (Supplementary Materials, Table S7). Intake was categorized based on quintiles, and a comparison was made between the highest quintile and the lowest. There were no significant changes in the observed risk estimates.

## Discussion

The outsized impact of CVD is primarily due to its asymptomatic nature during “silent” episodes and its progression to plaque deposits over time as blood vessels become blocked. Previous research has shown that the development of CVD is often accompanied by myocardial energy metabolism disorders [21]. Many clinical guidelines now recommend many alternative biomarkers [22]. The accuracy of the FRS, the most widely used tool [16], has been validated in a variety of populations [17, 18], and it is helpful for predicting CVD incidence and performing clinical primary prevention. For Chinese men, higher iron intake can reduce CVD risk, and the relationship was close to an inverse J-shape. These findings are similar to some previous findings [4]. Non-heme iron diet could reduce CVD risk [23]. In terms of dyslipidemia, a study in Tehran showed that total iron intake promoted high triglycerides [24]. However, other studies have not shown an influence on CVD mortality [25, 26] or lipids [6]. These study populations mostly originated from Western countries. These differences in results may be attributed to variations in ethnicity and diet.

For men, moderate heme iron intake could reduce CVD risk, and the effect was nearly U-shaped. However, higher heme iron intake increased high LDL-C, TC, TG, and low ApoA-1/ApoB risk and the relationship was close to an inverted L-shaped curve. Chen et al. [4] showed that less heme iron intake promoted nonfatal CVD incidence. However, some studies have come to different conclusions. When heme iron intake increases every 7 mg/day, CVD risk increases by 1% [24]. Heme iron intake promoted myocardial infarction but not stroke or CVD mortality [27]. This may be because Chinese individuals consume relatively little heme iron.

The relationships between total, non-heme iron intake and CVD incidence in women were close to L-shaped. Heme iron intake increased high LDL-C and high TC risk but did not reduce cardiovascular risk. This may be because men tend to retain excess iron [28], and women have less iron due to menstruation [29]. Heme iron intake promoted CVD in women. Moderate non-heme iron reduced CVD risk [30].

ApoB and ApoA-1 are the main surface proteins of atherosclerotic lipoproteins and HDL-C. A Swedish study showed that people with CVD had higher LDL-C levels and lower ApoA-1/ApoB ratios [31]. ApoA-1/ApoB is more precise than ApoB or ApoA-1 alone [32]. However, the measured levels of ApoB may not be consistent with those of LDL-C [33]. For individuals whose ApoB is highly inconsistent with LDL-C, the nutrients that contribute most significantly to the diet are total fat, saturated fatty acids, and thiamine [34]. Therefore, it is necessary to evaluate the levels of multiple lipid markers. The risk of a low ApoA-1/ApoB ratio gradually increased with increasing heme iron intake. The serum ferritin concentration increases the ApoB/ApoA-1 ratio [35], similar to this study.

### Study strengths and limitations

This study explored the nonlinear relationships of iron intake with CVD incidence and dyslipidemia in the Chinese population and comprehensively analyzed multiple risk factors, providing more reasonable dietary recommendations. Second, this study was rigorous in design and had a large sample size. Third, to minimize confounding effects, the model was adjusted for various factors.

This study has several limitations. First, long-term follow-up data were lacking. The study sample included only Chinese participants. Second, rough estimates of heme iron intake may lead to discrepancies. In addition, the CHNS does not include iron supplements or medications for lipid-lowering or blood control. However, studies have shown that the overall intake of dietary supplements among the Chinese population is relatively low, at approximately 0.71%. The intake of iron supplements is approximately 0.16% [36]. Therefore, the findings may not have changed materially. Third, some socioeconomic and behavioral factors were not considered in the present study, which may have confounded the results.

## Conclusions

In the process of preventing CVD risk and dyslipidemia, as well as formulating clinical management strategies, greater attention should be given to dietary iron intake. For men, moderate iron intake reduced CVD, low ApoA-1/ApoB, and high Lp (a) risk. Heme iron intake increased high LDL-C, TC, TG, and low ApoA-1/ApoB risks. Therefore, men, especially those at risk of developing dyslipidemia, should consume non-heme iron to prevent CVD. For women, moderate non-heme rather than heme iron intake can reduce CVD risk. Heme iron intake incresed high LDL-C and TC incidence. Therefore, women should minimize their heme iron intake to reduce dyslipidemia risk.

### Electronic supplementary material

Below is the link to the electronic supplementary material.

Supplementary Material 1

## Data Availability

The data supporting the findings of this study are available from CHNS (https://www.cpc.unc.edu/projects/china/data/datasets).

## Abbreviations

ApoA-1: Apolipoprotein A1
ApoB: Apolipoprotein B
BMI: Body mass index
BP: Blood pressure
CHNS: China Health and Nutrition Survey
CI: Confidence interval
CVD: Cardiovascular disease
FET: Ferritin
FRS: Framingham risk score
HDL-C: High-density lipoprotein cholesterol
LDL-C: Low-density lipoprotein cholesterol
Lp(a): Lipoprotein (a)
OR: Odds ratio
RCS: Restricted cubic splines
TC: Total cholesterol
TG: Triglyceride
TRF: Transferrin
TRFR: Transferrin receptor
