# Supplementary material for: Associations of dietary iron intake with cardiovascular disease risk and dyslipidemia among Chinese adults
Source: Lipids Health Dis. 2024 Mar 2;23:67. doi: 10.1186/s12944-024-02058-4 (PMC10908143; doi:10.1186/s12944-024-02058-4)
Supplement: Supplementary file 1 — Supplementary Material 1 [file 12944_2024_2058_MOESM1_ESM.docx]

Table S1 Comparative analysis between adult participants finally included vs excluded

|  | Participants finally included | Excluded adult participants | P value |
| --- | --- | --- | --- |
| Male |  |  |  |
| Age, years |  |  | 0.733 |
| <65 | 3289(87.0) | 6117(87.3) |  |
| ≥65 | 491(13.0) | 893(12.7) |  |
| Body mass index, kg/m^2^ |  |  | 0.790 |
| <20 | 1011(26.7) | 1893(27.0) |  |
| ≥20 | 2769(73.3) | 5117(73.0) |  |
| Smoke, n(%) | 1538(40.7) | 2953(42.1) | 0.154 |
| Drinking Alcohol, n(%) | 1517(40.1) | 2988(42.6) | 0.078 |
| urban |  |  |  |
| Female |  |  |  |
| Age, years |  |  | 0.153 |
| <65 | 3803（86.6） | 6317（85.6） |  |
| ≥65 | 590（13.4） | 1062（14.4） |  |
| Body mass index, kg/m^2^ |  |  | 0.725 |
| <20 | 1032（23.5） | 1711（23.2） |  |
| ≥20 | 3361（76.5） | 5668（76.8） |  |
| Smoke, n(%) | 170(3.9) | 177(2.4) | <0.001 |
| Drinking Alcohol, n(%) | 331(7.5) | 706(9.6) | 0.351 |
| urban |  |  |  |

*Variables are presented as the n (%).

Table S2 The description of missing data in covariates

| Variables | Non-missing | Missing |
| --- | --- | --- |
| Age | 8173 | 0 |
| BMI | 8074 | 99 |
| Alcohol status | 8172 | 1 |
| Smoking status | 8172 | 1 |
| Energy intake | 8173 | 0 |
| Urban residence | 8173 | 0 |
| Education level | 8044 | 129 |
| Ferritin | 8163 | 10 |
| Transferrin | 8165 | 8 |
| Transferrin receptor | 8163 | 10 |

Table S3 Population characteristics by quintiles of dietary heme and non-heme iron intake in man

| Variables* | Quintiles of heme iron intake,mg/day | | | | Quintiles of non-heme iron intake,mg/day | | | |
| --- | --- | --- | --- | --- | --- | --- | --- | --- |
|  | Q1 | Q3 | Q5 | *P* | Q1 | Q3 | Q5 | *P* |
| Age, years | 48.0(38.0,59.0) | 47.0(37.0,58.0) | 46.0(36.0,54.0) | <0.001 | 53.0(40.0,65.0) | 47.0(37.0,56.0) | 46.0(37.0,54.0) | <0.001 |
| Body mass index, kg/m^2^ | 21.5(19.9,23.5) | 21.3(19.9,23.6) | 21.5(19.7,23.8) | 0.133 | 21.5(19.7,23.6) | 21,5(19,9,23,7) | 21.4(19.8,23.6) | 0.605 |
| Systolic BP, mmHg | 119.3(108.0,125.0) | 118.0(106.7,125.0) | 115.0(107.3,124.0) | <0.001 | 120.0(108.7,130.0) | 116.0(108.7,124.0) | 115.0(106.5,122,0) | <0.001 |
| Diastolic BP, mmHg | 78.7(70.0,82.0) | 76.7(70.0,81.6) | 76.0(70.0,80.7) | 0.004 | 78.7(70.0,84.4) | 76.8(70.0,81.8) | 75.5(70.0,80.0) | 0.016 |
| Smoke, n(%) | 344(45.5) | 297(39.3) | 283(37.4) | 0.023 | 348(46.0) | 309(40.9) | 256(33.9) | <0.001 |
| Drinking Alcohol, n(%) | 188(24.9) | 210(27.8) | 217(28.7) | 0.172 | 224(29.6) | 220(29.1) | 174(23.0) | 0.017 |
| Diabetes, n(%) | 3(0.4) | 6(0.8) | 10(1.3) | 0.157 | 9(1.2) | 10(1.3) | 5(0.7) | 0.529 |
| Hypertension, n(%) | 33(4.4) | 31(4.1) | 33(4.4) | 0.938 | 42(5.6) | 33(4.4) | 31(4.1) | 0.113 |
| Urban Residence, n(%) | 112(14.8) | 236(31.2) | 361(47.8) | <0.001 | 271(35.8) | 246(32.5) | 225(29.8) | 0.089 |
| Education level, n(%) | | | | <0.001 |  | | | 0.055 |
| Primary school or lower | 441(58.3) | 340(45.0) | 267(35.3) |  | 371(49.1) | 341(45.1) | 327(43.3) |  |
| Middle school | 305(40.3) | 371(49.1) | 436(57.7) |  | 340(45.0) | 384(50.8) | 394(52.1) |  |
| college or above | 3(0.4) | 34(4.5) | 44(5.8) |  | 35(4.6) | 26(3.4) | 27(3.6) |  |
| Occupation, n(%) | | | | <0.001 |  | | | <0.001 |
| Farmer | 452(59.8) | 318(42.1) | 203(26.9) |  | 264(34.9) | 317(41.9) | 357(47.2) |  |
| Worker | 198(26.2) | 318(42.1) | 376(49.7) |  | 358(47.4) | 304(40.2) | 269(35.6) |  |
| Other | 106(14.0) | 120(15.9) | 177(23.4) |  | 134(17.7) | 135(17.9) | 130(17.2) |  |
| Dietary intake | | | | | | | | |
| Heme iron, mg/day | 0.2(0.0,0.2) | 0.9(0.8,1.0) | 2.2(1.9,2.9) | <0.001 | 0.7(0.3,1.1) | 0.9(0.5,1.6) | 1.0(0.5,1.8) | <0.001 |
| Nonheme iron, mg/day | 18.8(15.8,22.9) | 19.0(15.8,22.9) | 21.3(18.5,25.3) | <0.001 | 13.9(12.6,14.9) | 19.6(19.0,20.3) | 28.3(26.2,32.7) | <0.001 |
| Iron, mg/day | 18.9(16.0,23.0) | 19.8(16.7,23.8) | 23.9(20.9,28.3) | <0.001 | 14.7(13.4,15.7) | 20.7(19.8,21.5) | 30.0(27.5,34.3) | <0.001 |
| Energy, Kcal/day | 2725.6(2173.0,3257.2) | 2529.9(2073.5,3037.0) | 2540.8(2194.5,2999.1) | <0.001 | 2343.1(1863.6,2815.3) | 2544.7(2144.8,3035.1) | 2799.7(2361.9,3340.3) | <0.001 |
| Fat, g/day | 44.0(30.1,62.9) | 64.8(44.3,91.1) | 79.0(54.3,104.4) | <0.001 | 62.8(41.6,87.4) | 62.6(39.0,91.3) | 61.8(39.9,92.4) | 0.412 |
| Carbohydrate, g/day | 481.4(356.6,608.6) | 391.0(306.3,484.1) | 356.1(293.6,442.9) | <0.001 | 345.7(255.9,445.3) | 394.6(318.9,482.3) | 447.6(350.9,562.2) | <0.001 |
| Protein, g/day | 76.3(59.9,97.9) | 72.7(60.7,89.8) | 79.6(65.5,95.2) | <0.001 | 67.7(53.6,83.9) | 76.0(61.9,92.9) | 83.6(68.4,100.9) | <0.001 |
| Biomarkers | | | | | | | | |
| Ferritin, ng/ml | 110.0(70.3,192.2) | 120.7(72.8,217.9) | 135.6(76.3,244.0) | <0.001 | 111.1(68.1,199.4) | 124.6(72.8,217.4) | 114.2(70.8,199.9) | 0.007 |
| Transferrin, mg/dl | 279.0(249.8,315.0) | 273.0(244.0,310.0) | 276.5(249.0,311.0) | 0.005 | 272.0(240.0,311.0) | 276.0(246.0,306.0) | 280.0(250.8,315.0) | 0.035 |
| Transferrin receptor, mg/L | 1.3(1.1,1.5) | 1.3(1.1,1.6) | 1.3(1.1,1.6) | 0.211 | 1.3(1.1,1.6) | 1.3(1.1,1.6) | 1.3(1.1,1.6) | 0.002 |
| LDL-C, mmol/L | 2.8(2.3,3.3) | 2.8(2.3,3,4) | 2.9(2.4,3.6) | 0.004 | 2.9(2.3,3.6) | 2.9(2.3,3.4) | 2.8(2.3,3.4) | 0.106 |
| HDL-C, mmol/L | 1.3(1.1,1.6) | 1.3(1.1,1.6) | 1.3(1.1,1.6) | <0.001 | 1.3(1.1,1.6) | 1.3(1.1,1.6) | 1.3(1.1,1.6) | 0.003 |
| Total cholesterol, mmol/L | 4.6(4.0,5.2) | 4.7(4.1,5.3) | 4.9(4.3,5.5) | <0.001 | 4.8(4.2,5.4) | 4.7(4.2,5.4) | 4.7(4.1,5.4) | 0.575 |
| Triglycerides, mmol/L | 1.2(0.8,1.9) | 1.3(0.9,2.1) | 1.5(1.0,2.4) | <0.001 | 1.3(0.8,2.0) | 1.4(0.9,2.2) | 1.3(0.9,2.0) | 0.010 |
| Apolipoprotein A1, g/L | 1.1(0.9,1.2) | 1.1(0.9,1.3) | 1.1(0.9,1.3) | 0.012 | 1.1(0.9,1.3) | 1.1(0.9,1.3) | 1.1(0.9,1.3) | 0.439 |
| Apolipoprotein B, g/L | 0.8(0.7,1.0) | 0.9(0.7,1.1) | 0.9(0.8,1.1) | <0.001 | 0.9(0.7,1.1) | 0.9(0.7,1.1) | 0.9(0.7,1.1) | 0.258 |
| Lipoprotein (a), mg/L | 83.5(42.0,165.0) | 72.0(36.0,152.0) | 63.0(31.0,133.3) | <0.001 | 83.0(42.0,168.3) | 69.0(34.0,146.3) | 70.0(33.0,143.5) | <0.001 |

*Variables are presented as the mean (SD), median (IQR) or n (%).

Table S4 Population characteristics by quintiles of dietary heme and non-heme iron intake in woman

| Variables* | Quintiles of heme iron intake,mg/day | | | | Quintiles of non-heme iron intake,mg/day | | | |
| --- | --- | --- | --- | --- | --- | --- | --- | --- |
|  | Q1 | Q3 | Q5 | *P* | Q1 | Q3 | Q5 | *P* |
| Age, years | 49.0(38.0,60.0) | 47.0(36.0,58.0) | 45.0(35.0,54.0) | <0.001 | 51.0(39.0,65.0) | 47.0(37.0,56.0) | 45.0(37.0,54.0) | <0.001 |
| Body mass index, kg/m^2^ | 22.0(20.3,24.4) | 22.0(20.1,24.2) | 21.6(19.9,23.9) | <0.001 | 22.0(20.1,24.6) | 21.9(20.2,24.2) | 21.9(20.2,24.0) | 0.443 |
| Systolic BP, mmHg | 114.0(105.0,124.0) | 110.0(103.3,120.7) | 110.0(100.0,120.0) | <0.001 | 116.0(106.0,129.2) | 110.7(102.0,120.0) | 110.0(100.0,120.0) | <0.001 |
| Diastolic BP, mmHg | 75.0(70.0,80.0) | 73.3(68.0,80.0) | 72.0(66.7,80.0) | <0.001 | 76.0(70.0,81.3) | 73.3(68.0,80.0) | 70.7(66.7,80.0) | <0.001 |
| Smoke, n(%) | 51(5.8) | 33(3.8) | 16(1.8) | <0.001 | 48(5.5) | 33(3.8) | 21(2.4) | <0.001 |
| Drinking Alcohol, n(%) | 22(2.5) | 54(6.1) | 48(5.5) | 0.001 | 39(4.4) | 54(6.1) | 41(4.7) | 0.494 |
| Diabetes, n(%) | 6(0.7) | 10(1.1) | 7(0.8) | 0.527 | 15(1.7) | 0(0.0) | 4(0.5) | 0.001 |
| Hypertension, n(%) | 41(4.7) | 39(4.4) | 29(3.3) | 0.543 | 53(6.0) | 26(3.0) | 23(2.6) | <0.001 |
| Urban Residence, n(%) | 146(16.6) | 298(33.9) | 413(47.0) | <0.001 | 317(36.1) | 297(33.8) | 259(29.5) | 0.047 |
| Education level, n(%) | | | | <0.001 |  | | | 0.523 |
| Primary school or lower | 629(71.6) | 494(56.2) | 373(42.4) |  | 500(56.8) | 492(56.0) | 478(54.4) |  |
| Middle school | 224(25.5) | 336(38.2) | 461(52.4) |  | 335(38.1) | 346(39.4) | 374(42.5) |  |
| college or above | 7(0.8) | 27(3.1) | 35(4.0) |  | 25(2.8) | 25(2.8) | 13(1.5) |  |
| Occupation, n(%) | | | | <0.001 |  | | | <0.001 |
| Farmer | 529(60.2) | 396(45.1) | 262(29.8) |  | 286(32.5) | 397(45.2) | 466(53.0) |  |
| Worker | 269(30.6) | 364(41.4) | 412(46.9) |  | 435(49.5) | 355(40.4) | 289(32.9) |  |
| Other | 81(9.2) | 119(13.5) | 205(23.3) |  | 158(18.0) | 127(14.4) | 124(14.1) |  |
| Dietary intake | | | | | | | | |
| Heme iron, mg/day | 0.1(0.0,0.2) | 0.7(0.6,0.8) | 1.9(1.6,2.4) | <0.001 | 0.5(0.2,0.9) | 0.8(0.4,1.3) | 0.8(0.4,1.5) | <0.001 |
| Nonheme iron, mg/day | 16.2(13.4,19.9) | 16.9(14.0,20.1) | 18.7(16.3,22.3) | <0.001 | 12.1(11.0,13.0) | 17.1(16.6,17.7) | 25.1(23.0,29.0) | <0.001 |
| Iron, mg/day | 16.2(13.6,20.0) | 17.6(14.7,20.9) | 20.9(18.3,24.7) | <0.001 | 12.6(11.4,13.6) | 17.9(17.3,18.7) | 26.3(24.0,30.3) | <0.001 |
| Energy, Kcal/day | 2275.6(1800.3,2813.9) | 2221.8(1810.4,2597.3) | 2196.1(1809.7,2626.5) | 0.161 | 2004.7(1565.7,2436.8) | 2242.9(1873.6,2642.4) | 2427.0(2011.2,2912.7) | <0.001 |
| Fat, g/day | 40.1(26.4,57.4) | 59.2(42.1,84.2) | 70.3(48.5,96.0) | <0.001 | 54.1(37.0,80.7) | 58.5(37.6,83.7) | 55.2(37.9,81.3) | 0.049 |
| Carbohydrate, g/day | 400.1(303.8,525.8) | 337.9(262.7,420.8) | 316.0(241.6,387.0) | <0.001 | 299.6(218.8,386.0) | 342.1(275.1,424.9) | 383.9(297.9,494.5) | <0.001 |
| Protein, g/day | 63.3(48.8,81.2) | 63.8(51.9,77.3) | 69.1(57.2,84.3) | <0.001 | 57.6(46.1,71.8) | 66.2(54.4,80.4) | 71.4(59.2,86.9) | <0.001 |
| Biomarkers() | | | | | | | | |
| Ferritin, ng/ml | 56.5(26.6,103.4) | 53.4(25.9,94.7) | 47.3(22.4,87.4) | 0.458 | 53.9(24.6,93.3) | 49.1(23.8,93.7) | 51.9(25.2,99.9) | 0.013 |
| Transferrin, mg/dl | 292.0(262.0,333.0) | 288.0(253.0,327.0) | 283.0(253.0,318.0) | <0.001 | 282.0(252.0,318.0) | 291.0(262.0,329.0) | 291.0(257.0,333.0) | <0.001 |
| Transferrin receptor, mg/L | 1.4(1.1,1.7) | 1.4(1.1,1.7) | 1.4(1.1,1.7) | 0.586 | 1.4(1.1,1.8) | 1.4(1.1,1.7) | 1.4(1.2,1.7) | 0.974 |
| LDL-C, mmol/L | 2.9(2.4,3.6) | 2.9(2.4,3.6) | 3.0(2.4,3.6) | 0.312 | 3.0(2.4,3.7) | 2.9(2.4,3.6) | 3.0(2.4,3.6) | 0.326 |
| HDL-C, mmol/L | 1.4(1.2,1.7) | 1.4(1.2,1.7) | 1.5(1.2,1.7) | 0.526 | 1.5(1.2,1.7) | 1.4(1.2,1.7) | 1.5(1.3,1.7) | 0.149 |
| Total cholesterol, mmol/L | 4.8(4.1,5.5) | 4.8(4.2,5.5) | 4.8(4.2,5.6) | 0.115 | 4.9(4.3,5.6) | 4.8(4.2,5.5) | 4.8(4.2,5.6) | 0.137 |
| Triglycerides, mmol/L | 1.2(0.9,1.8) | 1.2(0.8,1.9) | 1.2(0.8,1.8) | 0.416 | 1.3(0.9,1.9) | 1.2(0.9,1.9) | 1.2(0.8,1.8) | 0.415 |
| Apolipoprotein A1, g/L | 1.1(1.0,1.3) | 1.1(1.0,1.3) | 1.2(1.0,1.4) | <0.001 | 1.1(1.0,1.3) | 1.1(1.0,1.3) | 1.1(1.0,1.3) | 0.041 |
| Apolipoprotein B, g/L | 0.9(0.7,1.0) | 0.9(0.7,1.1) | 0.9(0.7,1.1) | <0.001 | 0.9(0.7,1.1) | 0.9(0.7,1.1) | 0.9(0.7,1.1) | 0.152 |
| Lipoprotein (a), mg/L | 100.0(49.0,201.0) | 81.0(43.5,172.0) | 80.0(40.0,164.5) | <0.001 | 82.0(45.0,177.5) | 87.0(45.0,171.5) | 80.0(44.0,169.5) | 0.495 |

*Variables are presented as the mean (SD), median (IQR) or n (%).

Table S5 Association between iron intake and high cardiovascular disease risk and dyslipidaemia after exclusion of candidates with hypertension and diabetes.

| Dyslipidemia Type | Men(n=3604) | | | | | | Woman(n=4189) | | | | | |
| --- | --- | --- | --- | --- | --- | --- | --- | --- | --- | --- | --- | --- |
|  | heme iron intake | | | non-heme iron intake | | | heme iron intake | | | non-heme iron intake | | |
| High CVD risk |  |  |  |  |  |  |  |  |  |  |  |  |
| Q1 | Ref |  |  | Ref |  |  | Ref |  |  | Ref |  |  |
| Q2 | 1.089 | 0.804-1.473 | 0.582 | 0.657 | 0.499-0.865 | 0.003 | 0.996 | 0.679-1.463 | 0.986 | 0.681 | 0.256-1.810 | 0.441 |
| Q3 | 0.908 | 0.664-1.242 | 0.547 | 0.429 | 0.317-0.580 | <0.001 | 1.306 | 0.378-4.513 | 0.673 | 0.099 | 0.012-0.792 | 0.029 |
| Q4 | 0.830 | 0.598-1.152 | 0.266 | 0.286 | 0.203-0.402 | <0.001 | 0.919 | 0.228-3.699 | 0.906 | 0.347 | 0.092-1.314 | 0.119 |
| Q5 | 0.687 | 0.481-0.981 | 0.039 | 0.337 | 0.240-0.474 | <0.001 | 0.688 | 0.124-3.814 | 0.669 | 0.191 | 0.041-0.892 | 0.035 |
| High LDL-C |  |  |  |  |  |  |  |  |  |  |  |  |
| Q1 | Ref |  |  | Ref |  |  | Ref |  |  | Ref |  |  |
| Q2 | 1.241 | 0.837-1.840 | 0.283 | 1.088 | 0.770-1.535 | 0.633 | 1.193 | 0.872-1.632 | 0.270 | 1.259 | 0.925-1.712 | 0.143 |
| Q3 | 1.284 | 0.865-1.906 | 0.214 | 0.963 | 0.674-1.376 | 0.837 | 1.054 | 0.759-1.463 | 0.755 | 1.093 | 0.797-1.498 | 0.581 |
| Q4 | 1.621 | 1.100-2.390 | 0.015 | 0.710 | 0.484-1.040 | 0.079 | 1.629 | 1.189-2.232 | 0.002 | 1.273 | 0.925-1.750 | 0.138 |
| Q5 | 1.761 | 1.193-2.597 | 0.004 | 0.802 | 0.545-1.179 | 0.262 | 1.669 | 1.208-2.305 | 0.002 | 1.399 | 1.019-1.919 | 0.038 |
| Low HDL-C |  |  |  |  |  |  |  |  |  |  |  |  |
| Q1 | Ref |  |  | Ref |  |  | Ref |  |  | Ref |  |  |
| Q2 | 1.023 | 0.736-1.421 | 0.894 | 0.924 | 0.680-1.255 | 0.612 | 0.851 | 0.586-1.235 | 0.395 | 0.895 | 0.610-1.313 | 0.571 |
| Q3 | 1.090 | 0.787-1.512 | 0.603 | 0.894 | 0.656-1.218 | 0.477 | 0.737 | 0.500-1.087 | 0.124 | 1.045 | 0.716-1.527 | 0.818 |
| Q4 | 1.147 | 0.826-1.591 | 0.413 | 0.797 | 0.580-1.096 | 0.163 | 0.790 | 0.536-1.164 | 0.233 | 0.851 | 0.572-1.267 | 0.427 |
| Q5 | 1.247 | 0.897-1.732 | 0.189 | 0.688 | 0.490-0.965 | 0.030 | 0.744 | 0.495-1.119 | 0.155 | 0.741 | 0.490-1.122 | 0.157 |
| High TC |  |  |  |  |  |  |  |  |  |  |  |  |
| Q1 | Ref |  |  | Ref |  |  | Ref |  |  | Ref |  |  |
| Q2 | 1.561 | 0.992-2.455 | 0.054 | 0.958 | 0.648-1.415 | 0.829 | 1.247 | 0.897-1.734 | 0.189 | 1.260 | 0.916-1.733 | 0.155 |
| Q3 | 1.979 | 1.273-3.077 | 0.002 | 0.865 | 0.580-1.291 | 0.478 | 1.105 | 0.782-1.563 | 0.572 | 1.010 | 0.724-1.407 | 0.955 |
| Q4 | 2.145 | 1.372-3.352 | <0.001 | 0.828 | 0.553-1.240 | 0.359 | 1.837 | 1.322-2.553 | <0.001 | 1.314 | 0.946-1.827 | 0.104 |
| Q5 | 2.613 | 1.686-4.051 | <0.001 | 0.834 | 0.550-1.265 | 0.394 | 1.760 | 1.251-2.476 | 0.001 | 1.171 | 0.836-1.640 | 0.359 |
| High TG |  |  |  |  |  |  |  |  |  |  |  |  |
| Q1 | Ref |  |  | Ref |  |  | Ref |  |  | Ref |  |  |
| Q2 | 1.031 | 0.763-1.395 | 0.840 | 1.076 | 0.815-1.421 | 0.606 | 1.303 | 0.991-1.714 | 0.058 | 0.843 | 0.638-1.114 | 0.231 |
| Q3 | 1.472 | 1.097-1.975 | 0.010 | 1.030 | 0.778-1.364 | 0.835 | 1.277 | 0.964-1.693 | 0.088 | 1.095 | 0.836-1.435 | 0.509 |
| Q4 | 1.714 | 1.278-2.299 | <0.001 | 0.724 | 0.541-0.970 | 0.031 | 1.322 | 0.994-1.758 | 0.055 | 0.920 | 0.695-1.219 | 0.561 |
| Q5 | 2.005 | 1.494-2.689 | <0.001 | 0.720 | 0.533-0.972 | 0.032 | 1.343 | 0.999-1.806 | 0.050 | 0.803 | 0.600-1.074 | 0.139 |
| Low ApoA1/ApoB |  |  |  |  |  |  |  |  |  |  |  |  |
| Q1 | Ref |  |  | Ref |  |  | Ref |  |  | Ref |  |  |
| Q2 | 1.213 | 0.942-1.563 | 0.134 | 0.992 | 0.779-1.263 | 0.948 | 1.325 | 1.037-1.692 | 0.024 | 0.954 | 0.743-1.226 | 0.713 |
| Q3 | 1.325 | 1.028-1.707 | 0.030 | 0.920 | 0.720-1.176 | 0.506 | 1.173 | 0.911-1.511 | 0.216 | 1.039 | 0.809-1.333 | 0.765 |
| Q4 | 1.597 | 1.240-2.058 | <0.001 | 0.761 | 0.591-0.980 | 0.034 | 1.142 | 0.882-1.479 | 0.313 | 1.066 | 0.827-1.375 | 0.622 |
| Q5 | 1.493 | 1.153-1.933 | 0.002 | 0.887 | 0.688-1.145 | 0.358 | 1.216 | 0.934-1.583 | 0.147 | 1.246 | 0.967-1.606 | 0.089 |
| High Lp(a) |  |  |  |  |  |  |  |  |  |  |  |  |
| Q1 | Ref |  |  | Ref |  |  | Ref |  |  | Ref |  |  |
| Q2 | 0.869 | 0.632-1.195 | 0.387 | 1.157 | 0.857-1.560 | 0.341 | 0.758 | 0.576-0.999 | 0.049 | 1.508 | 1.143-1.991 | 0.004 |
| Q3 | 0.865 | 0.628-1.193 | 0.377 | 0.686 | 0.490-0.961 | 0.028 | 0.710 | 0.535-0.942 | 0.018 | 1.170 | 0.873-1.568 | 0.294 |
| Q4 | 1.055 | 0.769-1.447 | 0.740 | 0.911 | 0.660-1.257 | 0.570 | 0.903 | 0.685-1.191 | 0.471 | 1.211 | 0.901-1.628 | 0.205 |
| Q5 | 0.881 | 0.630-1.232 | 0.458 | 0.815 | 0.584-1.136 | 0.227 | 0.868 | 0.651-1.157 | 0.334 | 1.209 | 0.895-1.632 | 0.215 |

High CVD risk was adjusted by model 3.Dyslipidaemia risk was adjusted by Adjusted model.Mutual adjustment was performed for dietary heme iron and nonheme iron.

Intakes were estimated as energy-adjusted average intake from baseline

Table S6 Association between iron intake and high cardiovascular disease risk and dyslipidaemia after exclusion of candidates diagnosed with myocardial infarction.

| Dyslipidemia Type | Men(n=3772) | | | | | | Woman(n=4387) | | | | | |
| --- | --- | --- | --- | --- | --- | --- | --- | --- | --- | --- | --- | --- |
|  | heme iron intake | | | non-heme iron intake | | | heme iron intake | | | non-heme iron intake | | |
| High CVD risk |  |  |  |  |  |  |  |  |  |  |  |  |
| Q1 | Ref |  |  | Ref |  |  | Ref |  |  | Ref |  |  |
| Q2 | 0.778 | 0.403-1.500 | 0.454 | 0.717 | 0.412-1.249 | 0.240 | 0.790 | 0.410-1.524 | 0.482 | 0.721 | 0.414-1.254 | 0.246 |
| Q3 | 1.117 | 0.596-2.091 | 0.730 | 0.137 | 0.048-0.396 | <0.001 | 1.037 | 0.547-1.965 | 0.913 | 0.138 | 0.048-0.398 | <0.001 |
| Q4 | 0.824 | 0.413-1.644 | 0.583 | 0.631 | 0.332-1.200 | 0.160 | 0.845 | 0.423-1.687 | 0.633 | 0.553 | 0.283-1.080 | 0.083 |
| Q5 | 0.498 | 0.214-1.159 | 0.106 | 0.347 | 0.154-0.782 | 0.011 | 0.464 | 0.193-1.115 | 0.086 | 0.301 | 0.128-0.707 | 0.006 |
| High LDL-C |  |  |  |  |  |  |  |  |  |  |  |  |
| Q1 | Ref |  |  | Ref |  |  | Ref |  |  | Ref |  |  |
| Q2 | 1.239 | 0.917-1.673 | 0.162 | 1.124 | 0.841-1.501 | 0.430 | 1.229 | 0.910-1.659 | 0.178 | 1.119 | 0.838-1.495 | 0.446 |
| Q3 | 1.121 | 0.821-1.530 | 0.473 | 0.971 | 0.720-1.310 | 0.847 | 1.103 | 0.808-1.506 | 0.538 | 0.968 | 0.718-1.306 | 0.834 |
| Q4 | 1.669 | 1.234-2.256 | <0.001 | 1.115 | 0.826-1.506 | 0.475 | 1.648 | 1.219-2.229 | 0.001 | 1.092 | 0.808-1.476 | 0.567 |
| Q5 | 1.591 | 1.163-2.176 | 0.004 | 1.231 | 0.912-1.661 | 0.175 | 1.585 | 1.159-2.167 | 0.004 | 1.228 | 0.910-1.658 | 0.179 |
| Low HDL-C |  |  |  |  |  |  |  |  |  |  |  |  |
| Q1 | Ref |  |  | Ref |  |  | Ref |  |  | Ref |  |  |
| Q2 | 0.860 | 0.603-1.226 | 0.404 | 1.012 | 0.704-1.456 | 0.948 | 0.846 | 0.594-1.206 | 0.355 | 1.031 | 0.717-1.481 | 0.870 |
| Q3 | 0.723 | 0.498-1.050 | 0.088 | 0.951 | 0.652-1.388 | 0.795 | 0.715 | 0.493-1.038 | 0.078 | 0.953 | 0.653-1.390 | 0.802 |
| Q4 | 0.758 | 0.521-1.101 | 0.146 | 0.995 | 0.682-1.451 | 0.978 | 0.748 | 0.515-1.086 | 0.127 | 0.996 | 0.683-1.453 | 0.983 |
| Q5 | 0.683 | 0.460-1.016 | 0.060 | 0.781 | 0.522-1.169 | 0.230 | 0.675 | 0.455-1.003 | 0.052 | 0.783 | 0.523-1.173 | 0.236 |
| High TC |  |  |  |  |  |  |  |  |  |  |  |  |
| Q1 | Ref |  |  | Ref |  |  | Ref |  |  | Ref |  |  |
| Q2 | 1.204 | 0.872-1.661 | 0.259 | 1.164 | 0.859-1.578 | 0.328 | 1.203 | 0.873-1.659 | 0.259 | 1.156 | 0.854-1.565 | 0.348 |
| Q3 | 1.267 | 0.914-1.758 | 0.155 | 0.964 | 0.702-1.324 | 0.822 | 1.271 | 0.918-1.760 | 0.149 | 0.949 | 0.692-1.302 | 0.746 |
| Q4 | 1.870 | 1.361-2.571 | <0.001 | 1.153 | 0.840-1.581 | 0.378 | 1.855 | 1.351-2.548 | <0.001 | 1.140 | 0.831-1.563 | 0.417 |
| Q5 | 1.661 | 1.190-2.318 | 0.003 | 1.118 | 0.811-1.541 | 0.497 | 1.647 | 1.181-2.297 | 0.003 | 1.100 | 0.798-1.516 | 0.560 |
| High TG |  |  |  |  |  |  |  |  |  |  |  |  |
| Q1 | Ref |  |  | Ref |  |  | Ref |  |  | Ref |  |  |
| Q2 | 1.148 | 0.881-1.496 | 0.307 | 0.868 | 0.664-1.135 | 0.300 | 1.181 | 0.906-1.538 | 0.219 | 0.878 | 0.671-1.147 | 0.339 |
| Q3 | 1.205 | 0.920-1.577 | 0.175 | 1.016 | 0.780-1.323 | 0.907 | 1.218 | 0.931-1.595 | 0.150 | 1.027 | 0.789-1.338 | 0.842 |
| Q4 | 1.233 | 0.938-1.621 | 0.133 | 0.938 | 0.715-1.229 | 0.641 | 1.243 | 0.945-1.634 | 0.120 | 0.950 | 0.725-1.245 | 0.710 |
| Q5 | 1.222 | 0.919-1.625 | 0.168 | 0.810 | 0.611-1.073 | 0.141 | 1.231 | 0.925-1.637 | 0.154 | 0.814 | 0.614-1.078 | 0.151 |
| Low ApoA1/ApoB |  |  |  |  |  |  |  |  |  |  |  |  |
| Q1 | Ref |  |  | Ref |  |  | Ref |  |  | Ref |  |  |
| Q2 | 1.296 | 1.022-1.644 | 0.032 | 0.989 | 0.777-1.258 | 0.926 | 1.281 | 1.010-1.624 | 0.041 | 0.986 | 0.775-1.255 | 0.911 |
| Q3 | 1.190 | 0.932-1.519 | 0.162 | 1.002 | 0.785-1.278 | 0.988 | 1.172 | 0.918-1.495 | 0.202 | 1.001 | 0.785-1.277 | 0.993 |
| Q4 | 1.142 | 0.890-1.466 | 0.296 | 1.108 | 0.868-1.416 | 0.410 | 1.133 | 0.883-1.453 | 0.328 | 1.102 | 0.862-1.408 | 0.437 |
| Q5 | 1.163 | 0.900-1.504 | 0.248 | 1.226 | 0.958-1.569 | 0.106 | 1.144 | 0.885-1.478 | 0.305 | 1.226 | 0.958-1.569 | 0.106 |
| High Lp(a) |  |  |  |  |  |  |  |  |  |  |  |  |
| Q1 | Ref |  |  | Ref |  |  | Ref |  |  | Ref |  |  |
| Q2 | 0.760 | 0.580-0.995 | 0.046 | 1.392 | 1.056-1.833 | 0.019 | 0.762 | 0.581-0.998 | 0.048 | 1.389 | 1.055-1.830 | 0.019 |
| Q3 | 0.783 | 0.597-1.028 | 0.078 | 1.286 | 0.969-1.707 | 0.082 | 0.778 | 0.592-1.021 | 0.070 | 1.299 | 0.979-1.723 | 0.070 |
| Q4 | 0.905 | 0.690-1.186 | 0.467 | 1.312 | 0.985-1.746 | 0.063 | 0.905 | 0.691-1.186 | 0.470 | 1.289 | 0.967-1.717 | 0.083 |
| Q5 | 0.865 | 0.653-1.146 | 0.312 | 1.231 | 0.917-1.652 | 0.166 | 0.859 | 0.648-1.139 | 0.292 | 1.216 | 0.906-1.632 | 0.194 |

High CVD risk was adjusted by model 3.Dyslipidaemia risk was adjusted by Adjusted model.Mutual adjustment was performed for dietary heme iron and nonheme iron.

Intakes were estimated as energy-adjusted average intake from baseline

Table S7 Association between iron intake and high cardiovascular disease risk and dyslipidaemia after exclusion of candidates with BMI <18kg/m^2^ at baseline.

| Dyslipidemia Type | Men(n=3451) | | | | | | Woman(n=4127) | | | | | |
| --- | --- | --- | --- | --- | --- | --- | --- | --- | --- | --- | --- | --- |
|  | heme iron intake | | | non-heme iron intake | | | heme iron intake | | | non-heme iron intake | | |
| High CVD risk |  |  |  |  |  |  |  |  |  |  |  |  |
| Q1 | Ref |  |  | Ref |  |  | Ref |  |  | Ref |  |  |
| Q2 | 1.028 | 0.770-1.372 | 0.852 | 0.654 | 0.502-0.850 | 0.002 | 0.810 | 0.416-1.578 | 0.536 | 0.772 | 0.438-1.360 | 0.370 |
| Q3 | 0.842 | 0.625-1.134 | 0.259 | 0.457 | 0.344-0.608 | <0.001 | 1.125 | 0.592-2.139 | 0.720 | 0.148 | 0.051-0.428 | <0.001 |
| Q4 | 0.868 | 0.640-1.176 | 0.361 | 0.309 | 0.226-0.423 | <0.001 | 0.796 | 0.391-1.620 | 0.529 | 0.717 | 0.373-1.379 | 0.319 |
| Q5 | 0.722 | 0.522-1.000 | 0.050 | 0.362 | 0.263-0.499 | <0.001 | 0.512 | 0.218-1.203 | 0.124 | 0.383 | 0.169-0.871 | 0.022 |
| High LDL-C |  |  |  |  |  |  |  |  |  |  |  |  |
| Q1 | Ref |  |  | Ref |  |  | Ref |  |  | Ref |  |  |
| Q2 | 1.088 | 0.734-1.612 | 0.675 | 1.180 | 0.836-1.666 | 0.347 | 1.330 | 0.979-1.806 | 0.068 | 1.100 | 0.819-1.478 | 0.527 |
| Q3 | 1.243 | 0.845-1.829 | 0.269 | 1.005 | 0.700-1.442 | 0.981 | 1.148 | 0.834-1.580 | 0.397 | 0.961 | 0.709-1.303 | 0.796 |
| Q4 | 1.368 | 0.927-2.019 | 0.114 | 0.711 | 0.481-1.050 | 0.087 | 1.718 | 1.261-2.339 | <0.001 | 1.115 | 0.821-1.513 | 0.486 |
| Q5 | 1.727 | 1.180-2.529 | 0.005 | 0.824 | 0.556-1.219 | 0.333 | 1.637 | 1.190-2.252 | 0.002 | 1.242 | 0.917-1.683 | 0.161 |
| Low HDL-C |  |  |  |  |  |  |  |  |  |  |  |  |
| Q1 | Ref |  |  | Ref |  |  | Ref |  |  | Ref |  |  |
| Q2 | 1.057 | 0.762-1.466 | 0.741 | 0.908 | 0.669-1.233 | 0.537 | 0.895 | 0.622-1.288 | 0.549 | 0.975 | 0.671-1.418 | 0.895 |
| Q3 | 1.058 | 0.762-1.469 | 0.738 | 0.885 | 0.649-1.206 | 0.439 | 0.750 | 0.510-1.102 | 0.142 | 0.992 | 0.676-1.455 | 0.965 |
| Q4 | 1.138 | 0.819-1.582 | 0.441 | 0.743 | 0.538-1.024 | 0.070 | 0.816 | 0.556-1.196 | 0.297 | 0.901 | 0.607-1.339 | 0.606 |
| Q5 | 1.282 | 0.923-1.782 | 0.139 | 0.685 | 0.488-0.961 | 0.029 | 0.640 | 0.421-0.972 | 0.036 | 0.789 | 0.523-1.190 | 0.258 |
| High TC |  |  |  |  |  |  |  |  |  |  |  |  |
| Q1 | Ref |  |  | Ref |  |  | Ref |  |  | Ref |  |  |
| Q2 | 1.529 | 0.987-2.370 | 0.057 | 1.053 | 0.712-1.559 | 0.795 | 1.200 | 0.864-1.667 | 0.276 | 1.108 | 0.812-1.513 | 0.517 |
| Q3 | 1.916 | 1.250-2.937 | 0.003 | 0.962 | 0.644-1.439 | 0.852 | 1.287 | 0.923-1.794 | 0.136 | 0.939 | 0.680-1.297 | 0.704 |
| Q4 | 1.724 | 1.104-2.693 | 0.017 | 0.845 | 0.560-1.275 | 0.421 | 1.865 | 1.349-2.578 | <0.001 | 1.187 | 0.862-1.634 | 0.293 |
| Q5 | 2.351 | 1.531-3.609 | <0.001 | 0.896 | 0.588-1.364 | 0.608 | 1.690 | 1.207-2.367 | 0.002 | 1.129 | 0.816-1.562 | 0.463 |
| High TG |  |  |  |  |  |  |  |  |  |  |  |  |
| Q1 | Ref |  |  | Ref |  |  | Ref |  |  | Ref |  |  |
| Q2 | 1.133 | 0.839-1.531 | 0.414 | 1.072 | 0.810-1.417 | 0.628 | 1.215 | 0.927-1.592 | 0.158 | 0.885 | 0.674-1.162 | 0.379 |
| Q3 | 1.591 | 1.187-2.133 | 0.002 | 1.064 | 0.803-1.411 | 0.665 | 1.296 | 0.985-1.707 | 0.064 | 1.057 | 0.809-1.382 | 0.683 |
| Q4 | 1.702 | 1.265-2.288 | <0.001 | 0.722 | 0.538-0.970 | 0.031 | 1.368 | 1.035-1.808 | 0.027 | 0.909 | 0.689-1.200 | 0.502 |
| Q5 | 1.945 | 1.447-2.614 | <0.001 | 0.743 | 0.549-1.005 | 0.054 | 1.231 | 0.919-1.650 | 0.164 | 0.798 | 0.599-1.063 | 0.124 |
| Low ApoA1/ApoB |  |  |  |  |  |  |  |  |  |  |  |  |
| Q1 | Ref |  |  | Ref |  |  | Ref |  |  | Ref |  |  |
| Q2 | 1.177 | 0.915-1.514 | 0.204 | 1.018 | 0.800-1.296 | 0.884 | 1.382 | 1.083-1.764 | 0.009 | 0.995 | 0.777-1.276 | 0.971 |
| Q3 | 1.242 | 0.965-1.598 | 0.092 | 0.876 | 0.683-1.123 | 0.296 | 1.249 | 0.972-1.606 | 0.083 | 1.043 | 0.812-1.339 | 0.742 |
| Q4 | 1.399 | 1.085-1.805 | 0.010 | 0.709 | 0.549-0.916 | 0.009 | 1.198 | 0.927-1.549 | 0.167 | 1.153 | 0.896-1.482 | 0.269 |
| Q5 | 1.341 | 1.036-1.737 | 0.026 | 0.900 | 0.696-1.165 | 0.424 | 1.190 | 0.915-1.549 | 0.195 | 1.289 | 1.001-1.659 | 0.049 |
| High Lp(a) |  |  |  |  |  |  |  |  |  |  |  |  |
| Q1 | Ref |  |  | Ref |  |  | Ref |  |  | Ref |  |  |
| Q2 | 0.847 | 0.609-1.177 | 0.322 | 1.201 | 0.884-1.632 | 0.242 | 0.761 | 0.577-1.004 | 0.053 | 1.455 | 1.094-1.935 | 0.010 |
| Q3 | 0.815 | 0.584-1.139 | 0.231 | 0.627 | 0.439-0.895 | 0.010 | 0.758 | 0.572-1.004 | 0.053 | 1.251 | 0.930-1.684 | 0.139 |
| Q4 | 1.098 | 0.795-1.517 | 0.571 | 0.901 | 0.647-1.255 | 0.538 | 0.846 | 0.639-1.120 | 0.243 | 1.354 | 1.005-1.825 | 0.046 |
| Q5 | 0.891 | 0.631-1.256 | 0.509 | 0.797 | 0.565-1.126 | 0.199 | 0.820 | 0.613-1.096 | 0.180 | 1.285 | 0.948-1.742 | 0.107 |

High CVD risk was adjusted by model 3.Dyslipidaemia risk was adjusted by Adjusted model.Mutual adjustment was performed for dietary heme iron and nonheme iron.

Intakes were estimated as energy-adjusted average intake from baseline
